# Natural Relation of the Disease

**Published:** 1855-12

**Authors:** 


"*Natural Relation of the Disease.*—In the preliminary considerations of the nature of erysipelas I have already intimated my opinion, that its phenomena have marked analogy with those of the febrile exanthemata, and indicate a relation to gouty, rheumatic, and scorbutic inflammations in all of which the coetaneous inflammatory action of the internal mucous surface is a well-marked characteristic. All of them show an unmistakable tendency to a complication with gastric and biliary disorders, and are generally results of the same cold atmospheric humidity, from which an epidemic prevalence of all of them may proceed, as has been well marked during the present season. This relation of rheumatism and erysipelas will be yet more evident from the following comparison of the morbid state of the urine in both diseases, in which there is a very evident increase in the quantities of uric acid and extractive beyond a state of health. The results are indicated in the following tables, compiled from Simon's Chemistry:—

RHEUMATISM.

A man aged thirty, whose urine threw down a copious red sediment on standing for two hours, and was 1017.2 of specific gravity.

| | Analysis 1. | Analysis 2. | Analysis. 3. |
|---|---|---|---|
| Water, - - - - - | 971.80 | 970.20 | 981.10 |
| Solid constitutents, - - - | 28.20 | 29.8 | 18.90 |
| Urea, - - - - - - | 12.20 | 6.00 | 8.00 |
| Uric acid, - - - - - | 1.70 | 1.04 | 0.56 |
| Mixed salts, - - - | —— | 5.59 | 2.34 |
| Extractive matter, - - - | —— | 14.70 | 8.00 |

ERYSIPELAS.

In the febrile stage of this disease the urine has all the characters of inflammatory febrile urine. Becquerel made two qualitive analyses of the urine of a man, aged thirty-nine, who had erysipelas. Specific gravity, 1021 to 1023.1. The quantity passed during the twenty-four hours being, in the first and second analysis, 27.0 and 30.8 ounces respectively.

| | Analysis 1. | Analysis 2. | Analysis 3 of healthy urine. |
|---|---|---|---|
| Water, - - - - . | 965.5 | 961.9 | 981.0 |
| Solid constituents, - - - | 34.5 | 28.1 | 28. |
| Urea, - - - - - - | 12.5 | 12.7 | 12.1 |
| Uric acid, - - - - - | 1.2 | 1.3 | 0.4 |
| Fixed salts, - - - - | —— | 8.2 | 6.9 |
| Extractive matter, - - - | —— | 15.9 | 8.6 |

Other chemical pathologists have ascertained that, during the acute stage of erysipelas, while the urine presents all the characters of febrile urine, it is at the same time albuminous, and occasionally mixed with blood. These facts have been well established by the examinations of the urine, in erysipelatous cases, made by Becquerel and by Dr. Beguin, of Edinburgh. The presence also of albumen in the urine during the desquamative stage of erysipelas is noticed by Lehmann; and others have found that at this period of the disease, the urine is coagulable and charged with epithelium, as in scarlatina. The temporary albuminuria and the desquamation from the renal tubes, which have been thus found associated with erysipelas, and more particularly when the inflammation of the skin has been of great extent and idiopathic in its origin, are points of great practical importance in regard to this disease. They serve to show that this cutaneous affection, like other exanthematous diseases is associated with derangement of the renal functions, and that both are coetaneously connected with the same blood lesion the removal of which is an essential element of successful treatment. The efficacy of the tincture of sesquichloride of iron, which sensibly affects the secretion of the urine, while it restores the healthy nutritive powers of the red blood globules, probably depends on its power of producing these effects. The subject is one well worthy of further observation and experiment."—*London Lancet.*

